# Beyond the otoscope: an imaging review of congenital cholesteatoma

**DOI:** 10.1186/s13244-024-01761-1

**Published:** 2024-08-07

**Authors:** Guillaume Vangrinsven, Anja Bernaerts, Filip Deckers, Joost van Dinther, Andrzej Zarowski, Bert De Foer

**Affiliations:** 1 Departement of Radiology, ZAS Hospitals, Antwerp, Belgium; 2 Departement of Radiology, Antwerp University Hospital, Antwerp, Belgium; 3 European Institute for ORL-HNS, ZAS Hospitals, Antwerp, Belgium

**Keywords:** Congenital cholesteatoma, Temporal bone imaging, Conebeam computed tomography, Magnetic resonance imaging

## Abstract

**Abstract:**

Congenital cholesteatoma (CC) is a non-neoplastic lesion of keratin debris lined by epithelium found in the temporal bone. It is the lesser-known sibling of the acquired cholesteatoma and may be classified as congenital middle ear cholesteatoma and congenital petrous bone cholesteatoma. The incidence is rising, probably owing to increased recognition and advances in imaging modalities. Cone beam CT provides detailed anatomical information, highlighting quadrant location, ossicular involvement, and mastoid extension. MRI aids in lesion characterization and detection of complications. The classification systems for congenital middle ear and petrous bone cholesteatoma are helpful in the preoperative workup and have a role in predicting postoperative recurrence rates. Management almost invariably involves surgical intervention aimed at preserving middle and inner ear function. Follow-up of CC is mainly based on MRI together with otoscopic examination. Non-echo planar diffusion-weighted imaging, especially, has proven essential for detecting residual disease. This review article emphasizes the significance of imaging in the timely diagnosis and management of CCs.

**Clinical relevance statement:**

This article underscores the crucial role of imaging for prompt detection, preoperative assessment, and postoperative follow-up of CCs, a condition with rising incidence associated with potentially severe complications.

**Key Points:**

- Timely diagnosis of CCs is imperative for avoiding complications.
- Imaging is key in detection, preoperative evaluation, and postoperative management.
- Cone Beam CT and non-echo planar DWI represent state-of-the-art imaging techniques.

**Graphical Abstract:**

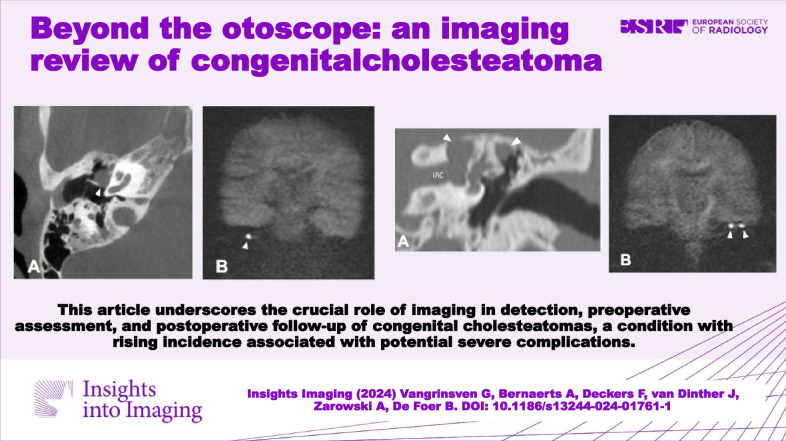

## Introduction

Cholesteatoma is defined by the European Academy of Otology and Neurotology and the Japanese Otological Society as a mass formed by the keratinizing squamous epithelium in the tympanic cavity and/or mastoid and subepithelial connective tissue and by the progressive accumulation of keratin debris with or without a surrounding inflammatory reaction [1]. They are histologically almost identical to the epidermoid cysts found in the cerebellopontine angle and the epidermoid inclusion cysts found in the skin. Cholesteatomas can be subdivided into two broad categories: congenital and acquired. Opposed to acquired cholesteatoma (AC), congenital cholesteatomas (CC) were thought to be very rare during the 19th and 20th centuries [2]. In 1965 Derlacki and Clemis defined CC as a cholesteatoma that develops behind an intact tympanic membrane in a patient without a previous history of aural infections [3]. It was later proposed that CC could not be excluded solely on grounds of prior bouts of otitis media and this definition is still in use today [4]. CC can be further subdivided into congenital middle ear cholesteatoma (CMEC) and congenital petrous bone cholesteatoma (CPBC), the latter arising outside the tympanic cavity. It is important to recognize that there is some overlap between these two categories as the intratympanic lesions may show extratympanic extension and vice-versa. CMEC and CPBC share similar histologic and imaging characteristics and from a radiological perspective, it therefore seems logical to focus on all CCs arising within the temporal bone.

## Etiology

CMECs are generally considered truly congenital, and unlike AC, they are not associated with Eustachian tube dysfunction or tympanic membrane retraction. The “epidermoid formation” theory is one of the most prominent theories regarding its origin and is based upon the discovery of a group of non-keratinizing epidermal cells in the embryonic development of the middle ear. Further growth of this formation could explain why most CCs occur in the anterior-superior quadrant [5–7]. Other theories include failure in a barrier function of the tympanic ring with migration of ectodermal tissue into the middle ear cleft or implantation of epithelial cells on the malleus handle due to tympanic retraction [8, 9].

While poorly studied, CPBCs are believed to originate from misplaced ectodermal cells during early embryonic development, potentially sharing a similar origin with intradural epidermoid cysts [10]. Histological studies have proven the existence of an organ in certain fish and birds which originates from ectodermal cells in close proximity to the geniculate ganglion and this entity has also been described as a vestigial structure in human embryos. This is an intriguing yet unproven theory regarding the origin of supralabyrinthine CPBC, especially considering its frequent association with the geniculate ganglion region [5, 11].

It’s important to note that none of these theories provide a definitive explanation for the origin of all CC. It is more likely that different lesions can arise through different mechanisms or even a combination of different mechanisms.

## Clinical features

CMEC is rare, and its exact incidence and prevalence are unknown due to a significant number of asymptomatic cases and sometimes difficult differentiation between congenital and ACs. Originally believed to be even rarer, the incidence of CC has been increasing since the mid-90s. The incidence of CMEC was estimated at 0.12 per 100,000 (or roughly 1 every million) person-years according to one surgical study and about 1–5% of all middle ear cholesteatomas are of the congenital type [9, 12]. CMEC, just like acquired middle ear cholesteatoma, is more common in boys than girls with a ratio of 3:1 and the mean age at diagnosis is 6–7 years [13–15]. Both ears are equally affected and bilateral CMEC is possible but very rare.

The classical presentation of a CMEC is that of a pearly white mass behind an intact tympanic membrane. The existing literature shows that 23–30% of patients have a history of inflammatory disease of the middle ear but in most patients, the inflammation is not a prominent feature [13, 16]. Conductive hearing loss is the major presenting symptom while sensorineural hearing loss, otalgia, facial nerve dysfunction, and tinnitus are less common [13, 16, 17].

Data on CPBC are rather limited. It is a rare disease and about 12–15% of all petrous bone cholesteatomas are of the congenital type [11, 18]. Mean age at diagnosis is higher for CPBC compared to CMEC, around 38–46 years [11, 18–20]. This later presentation may be due to the paucity of symptoms as well as the inability to detect most lesions on otoscopy [21]. Just like CMEC there is no clear right-left predilection and bilateral lesions are very rare.

Mixed hearing loss is the major presenting symptom in CPBC. Facial nerve dysfunction is a fairly typical presentation most commonly seen in supralabyrinthine disease [11, 19, 22]. Vertigo, otorrhea, and otalgia are less common. Other, rare complications include abscesses, labyrinthitis, sinus thrombophlebitis, and meningitis.

## Congenital cholesteatoma staging

### Congenital middle ear cholesteatoma

For staging of CMEC the middle ear is often divided into quadrants defined as follows: a vertical axis runs through the handle of the malleus and a horizontal axis, perpendicular to the vertical axis, runs through the umbo (Table 1). Over 80% of cases of CMECs involve the anterior-superior quadrant and this is often considered the site of origin [14, 23–25]. Larger lesions will typically extend posteriorly and superiorly into the posterior-superior quadrant, towards the incudostapedial joint, and between the malleus and incus [23]. This growth pattern is important because of frequent ossicular erosion when the cholesteatoma reaches the ossicular chain [23, 26]. Superior growth is bounded by the attic while medial and lateral growth is limited by the otic capsule and eardrum respectively. In more advanced cases the mastoid will also be involved, similar to acquired middle ear cholesteatomas [16]. Invasion of the inner ear is rare and lateral growth with rupture of the eardrum could represent end-stage disease [27]. Interestingly, in the Asian population predominant involvement of the posterior-superior quadrant has been reported, which could point to a different growth pattern or a different etiopathogenesis altogether [28].

**Table 1:** Potsic staging of congenital middle ear cholesteatomas

Potsic staging of congenital middle ear cholesteatoma	
Stage 1	
• Single quadrant involed	
• No ossicular involvement	
• No mastoid extension	
Stage 2	
• Multiple quadrants involved	
• No ossicular involvement	
• No mastoid extension	
Stage 3	
• Ossicular involvement^a^	
• No mastoid extension	
Stage 4	
• Extension into the mastoid	

The right column features schematic illustrations of a left tympanic membrane as observed during otoscopy. The green dotted lines represent the subdivision of the middle ear into four quadrants and the red area represents the CC. Ossicular erosion (stage 3) most often involves the long process of the incus and incudostapedial joint

^a^ Defined as: ossicular erosion and/or resection of one or more ossicles during surgery

The most widely used classification for CMEC was created by Potsic in 2002 (Table 1) [24]. This 4-stage classification system was primarily developed for intra-operative risk assessment of residual disease after surgery. It is, however, applicable to imaging and especially CT. The risk of postoperative residual cholesteatoma increases with higher disease grade (13% and 67% for stage 1 and stage 4 disease, respectively) and the main predictors for residual disease are ossicular involvement and mastoid extension of the disease [24]. In the same year, another article by Nelson et al presented an abbreviated version of the classification, omitting Potsic stage 2 [26]. Studies have shown that Potsic stage 2 disease is relatively uncommon and probably clinically not relevant [29].

CMEC has also been subdivided into closed-type (round epithelial cyst, most common) and open-type (keratinizing epithelium without formation of an epithelial cyst) based on histopathology or intra-operative findings. Open-type cholesteatomas are correlated with higher postoperative recurrence rates [30, 31].

### Petrous bone cholesteatoma

Staging of petrous bone cholesteatoma is based on the presumed initial site of the lesion and its extension without differentiation between etiologies. Sanna et al published their classification of petrous bone cholesteatoma in 1997 and it is the most widely used classification to our knowledge (Table 2). The goal is to help in surgical planning based on radiological findings. Class I (supralabyrinthine) petrous bone cholesteatoma is the most common location of congenital and ACs and makes up about half the cases. These lesions are located above the bony labyrinth and are very often in close relationship with the geniculate ganglion or the tympanic segment of the facial nerve [11, 18, 20]. Posterior extension may involve the posterior bony labyrinth, inferior extension may involve the cochlea and medial extension may involve the internal auditory canal (IAC) and petrous apex. Class II (infralabyrinthine) lesions are located beneath the bony labyrinth and may extend up to the internal carotid artery anteriorly, the posterior cranial fossa posteriorly, the IAC and clivus medially and the jugular bulb and lower cranial nerves inferiorly. When an infralabyrinthine cholesteatoma extends anteriorly past the internal carotid artery and into the petrous apex it becomes a class III infralabyrinthine-apical lesion. Class IV (massive) is defined by the involvement of the entire otic capsule with combined supra- and infralabyrinthine disease. Class V cholesteatomas are isolated to the petrous apex and are generally considered congenital [32, 33].

**Table 2:** Classification of petrous bone cholesteatomas as presented by Sanna et al in 1993

Sanna classification of petrous bone cholesteatomas	
Class	Location
Class I: Supralabyrinthine	Above the bony labyrinth
Class II: Infralabyrinthine	Beneath the bony labyrinth
Class III: Infralabyrinthine-apical	Beneath the bony labyrinth extending up to the petrous apex
Class IV: Massive	Involving the entire otic capsule
Class V: Apical	Isolated to the petrous apex

This classification is based on the location and extension of the lesion in the temporal bone. The classification does not differentiate between different etiologies

## Congenital cholesteatoma imaging

### Imaging techniques

Computed tomography (CT) is often the initial imaging modality for conductive hearing loss. Its excellent bone definition and high-resolution help accurately locate lesions and evaluate extension [34]. Cone beam computed tomography (CBCT) has several advantages over multidetector computed tomography (MDCT). CBCT provides a superior spatial resolution, will generally show less beam hardening artifacts but it is subject to motion artifacts [35]. Various studies have shown that CBCT delivers lower radiation doses than MDCT. This difference depends greatly on the type of scanner, scanner settings, and patient-specific factors. Furthermore, low-dose MDCT protocols have significantly advanced over the last decade, narrowing the gap [36–40]. Slice thickness should be kept as low as possible (for CBCT ideally 0.15 mm) without gaps and a field of view of 15 × 5 cm, with a separate small field of view reconstruction centered on each ear afterward. CBCT images can be reformatted in any plane without loss of resolution. This is particularly interesting since the evaluation of the temporal bone often requires reconstructions in specific planes [41]. Axial images are generally oriented parallel to the lateral semicircular canal with coronal images perpendicular to the plane of the lateral semicircular canal. Double-oblique reconstructions allow for detailed visualization of the incudostapedial joint and stapes superstructure. Reconstructions in Poschl and Stenvers plane (as well as reconstructions through the other semicircular canals) can be used when there is suspected semicircular canal dehiscence or fistulation. Additional non-standard reconstruction can also be made for better evaluation of the cholesteatoma and its relation to surrounding structures.

**Fig. 1:**
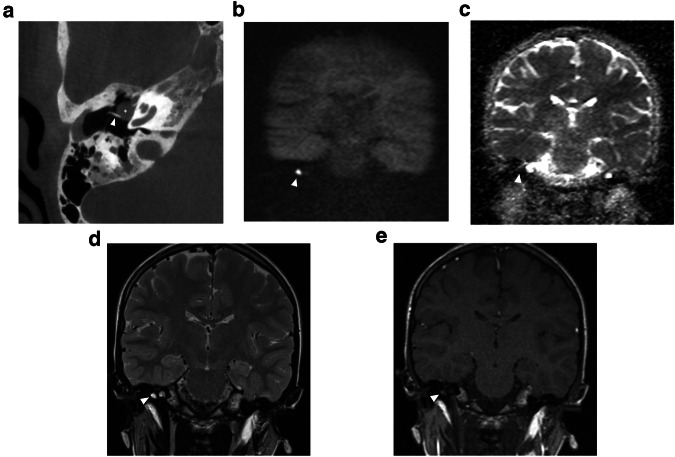
Eleven-year-old boy with conductive hearing loss in the right ear. **a** Axial CBCT image of a congenital middle ear cholesteatoma (CMEC) presenting as a well-rounded intratympanic mass in the anterior-superior quadrant (asterisk) and expanding medial to the malleus handle (arrowhead in **a**). **b** Non-EP DWI b1000, (**c**) calculated ADC map, (**d**) coronal TSE T2-weighted image, and (**e**) coronal TSE T1-weighted image: the white arrowhead points to the same lesion in these four images. The lesion has a high signal intensity on DWI and a low signal intensity on the ADC map. There is an intermediate signal intensity on T2-WI and a low signal intensity on T1-WI. The signal characteristics are almost pathognomonic of a cholesteatoma, in this case, a congenital cholesteatoma (CC)

**Fig. 2:**
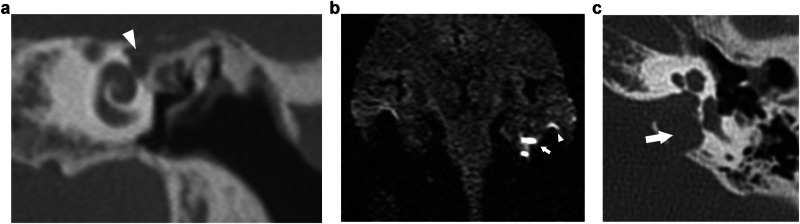
Fifty-five-year-old male with long-standing left-sided deafness, now presenting with a sudden onset of facial nerve paralysis. Supralabyrinthine (Class I) congenital petrous bone cholesteatoma (CPBC) in the left temporal bone. Coronal (**a**) and axial (**b**) computed tomography (CT) images of the left temporal bone show a well-circumscribed lytic lesion in the supralabyrinthine region with involvement of the geniculate ganglion (arrowhead in **a**), posterior extension medial to the vestibulum and dehiscent internal auditory canal (IAC) (arrow in **b**). There is high signal intensity in the medial petrous bone on EP DWI (b1000) (**c**) partially corresponding to the known cholesteatoma (arrow in **c**). There is a distorted aspect of the cholesteatoma making it less conspicuous. EP DWI is more susceptible to artifacts, mainly between the temporal bone and brain tissue (arrowhead in **c**), and has a lower in-plane resolution compared to non-EPI (compared to the DWI in Fig. 6)

Magnetic resonance imaging (MRI) has gained an important role in cholesteatoma imaging. Although spatial resolution is not comparable to CT, it has a higher specificity in the diagnosis of cholesteatoma. MRI protocols should include non-echo planar (EP) diffusion-weighted imaging (DWI) with apparent diffusion coefficient (ADC) maps, T2-weighted images (T2-WI), and T1-weighted images (T1-WI) without contrast administration. These three sequences form the cornerstones of current cholesteatoma imaging on MRI (Fig. 1). Originally, EP-DWI showed promising results in the diagnosis and postoperative follow-up of cholesteatoma [42]. But this sequence is subject to susceptibility artifacts at the air-bone interfaces due to heavy T2*-weighting. This makes the sequence less reliable for cholesteatoma detection, especially for small cholesteatoma inferior to 5 mm (Fig. 2) [43, 44]. Non-EP DWI sequences are less vulnerable to susceptibility artifacts and distortion and are now considered state-of-the-art. Non-EP DWI also allows higher in-plane resolution with thinner image slices [44, 45]. Non-EP DWI should be acquired with a b-factor of 0 (or 50) sec/mm^2^ and 1000 sec/mm^2^ with calculated ADC maps which help differentiate true diffusion restriction seen in cholesteatomas from other causes of high signal intensity on b1000 DWI [46]. To further enhance diagnostic confidence and lesion location some authors advocate the use of an additional DWI sequence in the axial plane (often EP-DWI) and fusion images of both DWI and 3D heavily T2-WI [47].

### Imaging findings

**Fig. 3:**
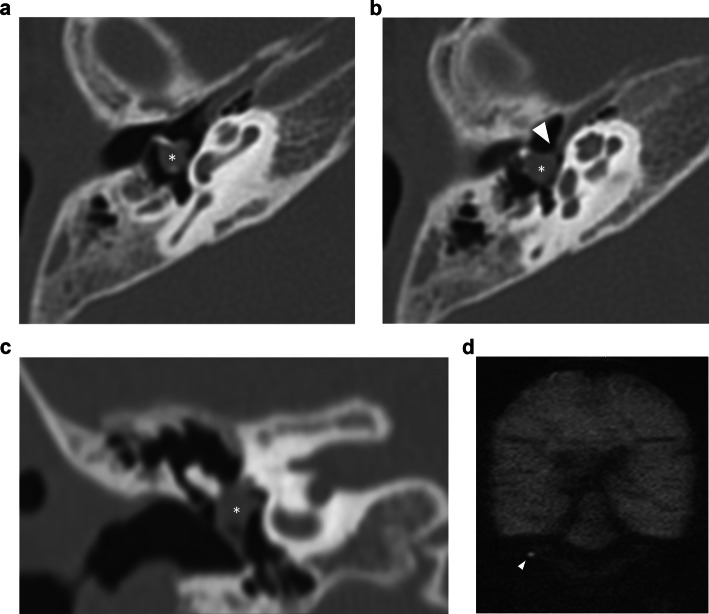
Five-year-old boy with conductive hearing loss (45 dB) on the right side. **a**, **b** Axial CT image of the right temporal bone shows a sharply delineated nodular mass lesion compatible with a CC (asterisks) growing between the malleus handle and long process of the incus and reaching the tensor tympani tendon superiorly (arrowhead). **c** Normal appearing scutum and Prussak’s space on this coronal reformatted CT image of the right temporal bone. Note lysis of the lenticular process of the incus which should be visible on this slice, corresponding to a Potsic stage 3. **d** Lesion correlating with a hyperintense focus on non-EP DWI sequence, confirming the diagnosis of a CC

**Fig. 4:**
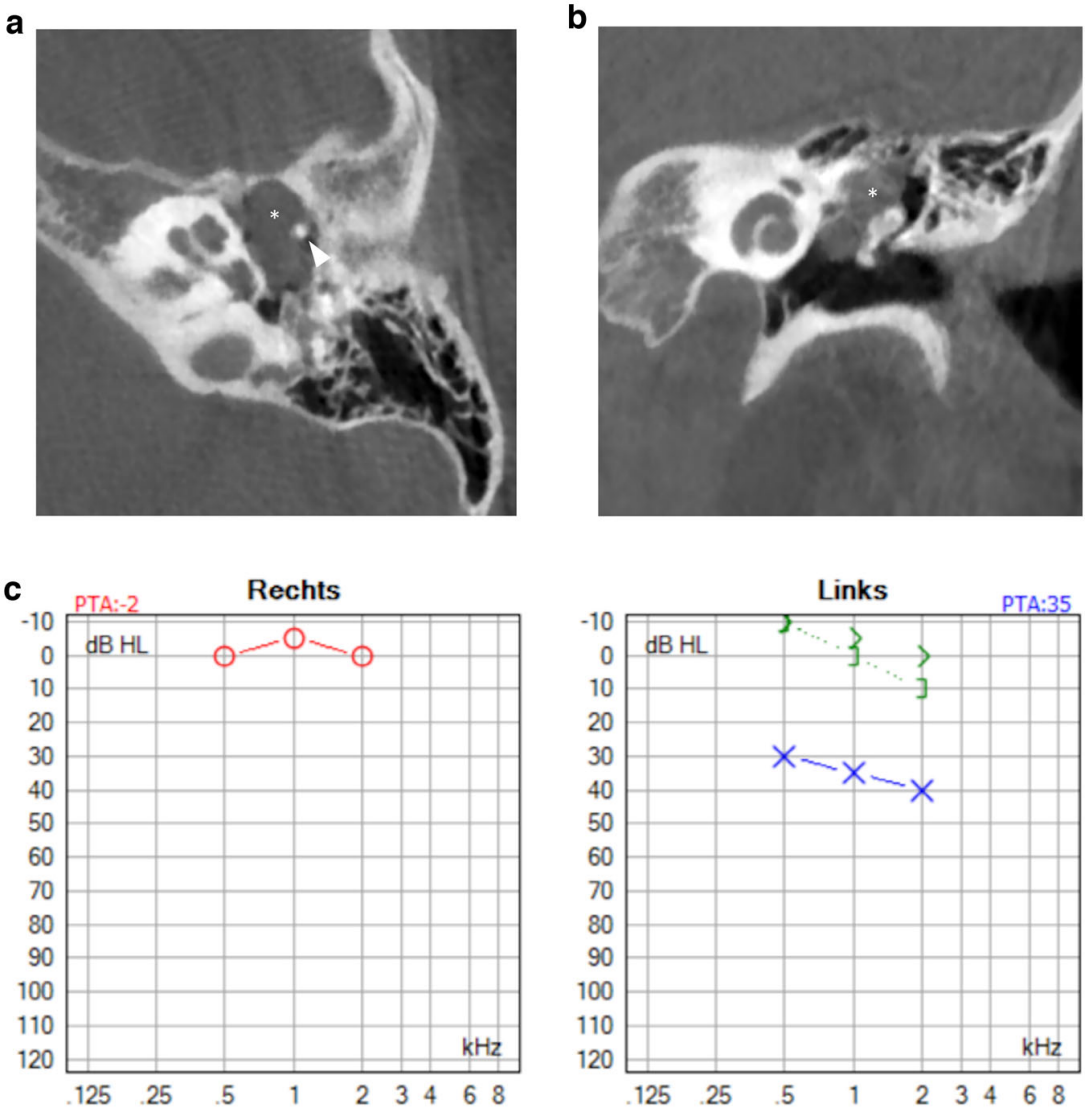
Eight-year-old boy with progressive conductive hearing loss and an intact tympanic membrane on the left side. MRI of the posterior fossa (images not shown) demonstrated a lesion in the left middle ear with restricted diffusion, compatible with a (congenital) cholesteatoma. Axial (**a**) and coronal (**b**) CBCT of the left temporal bone. Sharply delineated soft tissue mass (asterisks) filling the anterior-superior and posterior-superior quadrant with intact scutum compatible with a CMEC. Notice how the lesion grows medial of the malleus (arrowhead in **a**) and has eroded the long process of the incus, which should be visible on this axial slice. Note the minor motion artifacts. **c** Tone audiometry of the right (“rechts”) and left (“links”) ear. Normal hearing on the right side. The left ear shows diminished air conduction (30–40 dB) compared to the normal bone conduction (air-bone gap) indicating conductive hearing loss

**Fig. 5:**
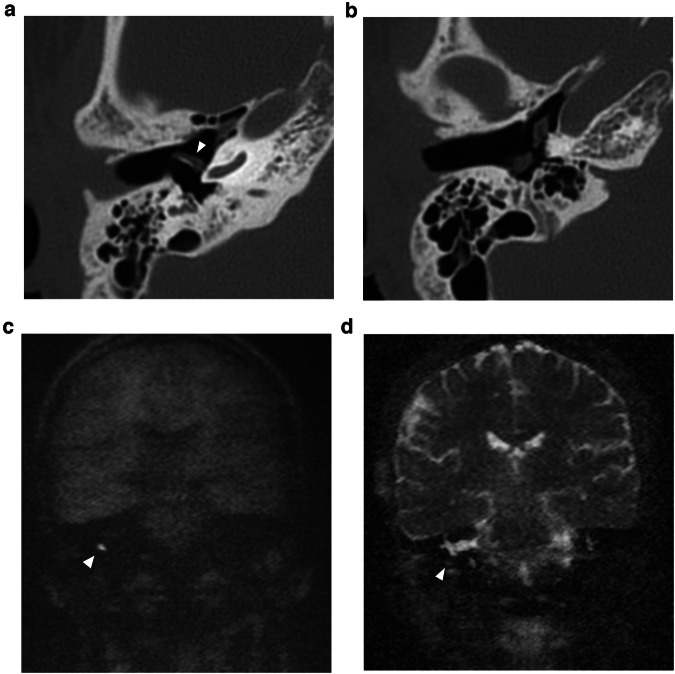
Sixteen-year-old male patient with conductive hearing loss on the right. **a**, **b** CBCT showing an irregular and discontinuous soft tissue lesion growing along the malleus handle and in the mesotympanum. Diffusion restriction on the b1000 DWI (**c**) and corresponding ADC map (**d**), in keeping with a CC. The growth pattern is suggestive of an “open-type” morphology

On CT, CMEC most often manifests as a relatively well-defined round or oval soft tissue mass in the tympanic cavity (Figs. 1, 3, and 4). The most common location is the anterior-superior quadrant, often near the tensor tympani tendon [16, 27, 48]. The cochleariform process has been proposed as a landmark to define extension in the posterior-superior quadrant [49]. CC growing in the posterior-superior quadrant will abut and later erode the incudostapedial joint (corresponding to Potsic stages 2 and 3, respectively). The long process of the incus is most commonly eroded followed by erosion of the stapes, classically sparing the stapes footplate, while erosion of the malleus is uncommon [23, 26, 29]. Erosion of the long process of the incus is often readily visible on axial imaging, but the erosion of the lenticular process and stapes superstructure can be subtle and is better appreciated on double-oblique and coronal reconstructions. CMEC can extend superiorly into the anterior epitympanic recess and posteriorly in the facial recess and tympanic sinus (Fig. 4). More advanced cases will show involvement of the mastoid antrum and mastoid air cells (Potsic stage 4) [16]. It is important to systematically report these extensions because they can be difficult to reach surgically and can affect operative management. Typical, well-defined, and rounded cholesteatomas correlate with closed-type CC while open-type cholesteatomas will often exhibit a more irregular and elongated shape (Fig. 5) [31].

**Fig. 6:**
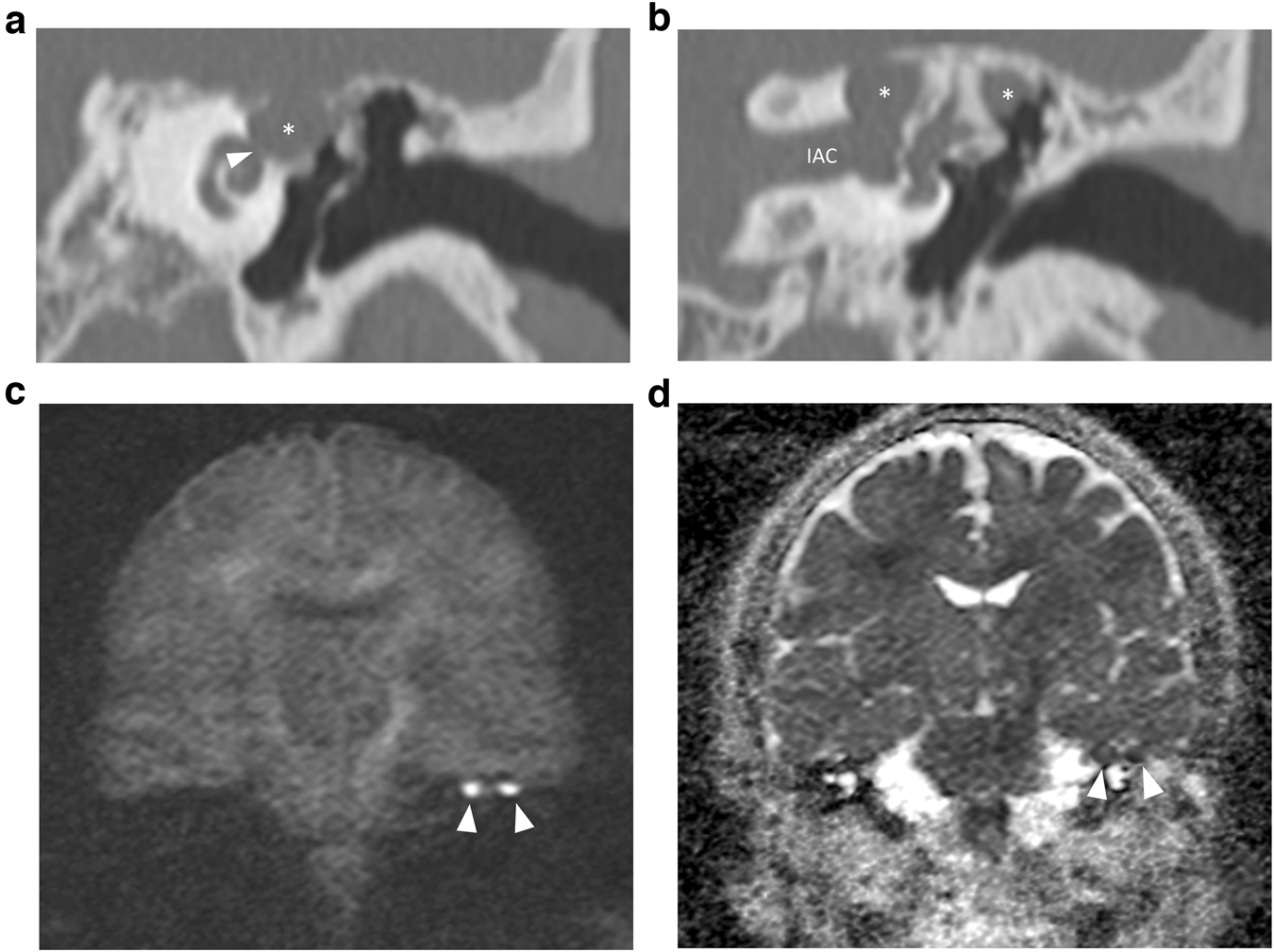
Supralabyrinthine (Class I) CC of the left temporal bone in a 57-year-old female patient presenting with peripheral facial paresis and mixed hearing loss on the left side. Coronal reformatted CT images of the left temporal bone show a CC in the supralabyrinthine region in close relation to the geniculate ganglion (asterisk in **a**) and dehiscence of the middle cochlear turn (arrowhead in **a**). The CC extends further backward, medial, and lateral to the superior semicircular canal (asterisks in **b**) with a large dehiscence of the IAC. Non-EP DWI and ADC map (**c**, **d**) at the same level as (**b**) show true restricted diffusion consistent with the diagnosis of a CC (arrowheads in **c**, **d**)

**Fig. 7:**
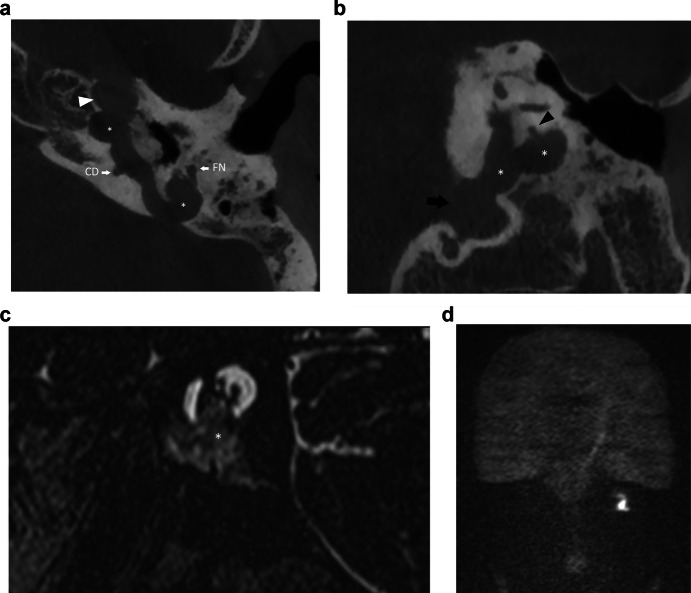
Forty-eight-year-old man with an infralabyrinthine (Class II) CPBC in the left temporal bone. Axial (**a**) and coronal (**b**) CBCT images of the left temporal bone show a well-circumscribed C-shaped lytic lesion in the infralabyrinthine region (asterisks in **a**, **b**). The lesion has eroded the skull base down to the sigmoid sinus inferiorly (black arrow in **b**) and the carotid canal anteriorly (white arrowhead in **a**). Posteriorly there is a dehiscence of the posterior fossa and of the posterior semicircular canal (black arrowhead in **b**). Sagittal heavily T2-WI (**c**) displays the growth in the IAC and possible dehiscence to the cochlea (asterisk). There is high DWI signal intensity in the infralabyrinthine petrous bone on non-EP DWI (b1000) (**d**) compatible with CC. CD, cochlear duct; FN, facial nerve

CPBC presents as a lytic lesion with soft tissue density and sharply defined borders embedded in the petrosal bone. CC matrix does not calcify. It is not uncommon to see large lesions at presentation because of limited symptoms and subsequent diagnostic delay. As already mentioned, a connection with the geniculate ganglion is almost invariably present in supralabyrinthine lesions (Figs. 2, 6) [19]. CT is key in the preoperative evaluation of the extent and complication of CPBC. Supra- and infralabyrinthine cholesteatomas can erode the bony outline of the cochlear basal and sometimes middle turn (Figs. 6, 7). This has a poor prognosis with regard to postoperative hearing and should be reported. IAC dehiscence can be present in both supra- and infralabyrinthine lesions and increases the risk for postoperative CSF leaks. Dehiscence of the semicircular canals, internal carotid artery, sigmoid sinus, and jugular bulb are important findings for the surgeon that can be suggested based on the imaging findings.

On MRI, the smallest lesion that can be detected on non-EP DWI is around 2 mm [50, 51]. Cholesteatomas are very bright on DWI, the so-called “light bulb” appearance. The signal intensity should be low on the ADC maps corresponding to true restricted diffusion (Fig. 1). A potential pitfall is the absence of the internal matrix with a lack of diffusion restriction (e.g., due to spontaneous evacuation), but in our experience, this is less common in CC compared to AC. Cholesteatomas characteristically present with an intermediate signal on T2-WI, generally lower than inflammatory changes or fluid. T2-WI will also help locate the lesion within the temporal bone, especially when acquired in two perpendicular planes. On T1-WI lesions have a low signal intensity, iso-intense to the cerebral cortex. Delayed gadolinium-enhanced T1-WI does not yield any clear diagnostic advantage over non-EP DWI for diagnosis of ACs and the same is probably true for CC [52]. In selected cases, contrast-enhanced T1-WI may be helpful when searching for alternative diagnoses. MRI is also useful in the detection of complications. Fistulation or invasion of the semicircular canals or basal turn of the cochlea can usually be detected on T2-WI but this is better appreciated on 3D heavily T2-WI of the fossa posterior and form an exception to administer IV-gadolinium in order to evaluate potentially associated active labyrinthitis. These are important to report because of the associated risk of postoperative hearing and vestibular dysfunction. Invasion of the IAC or intracranial compartment is usually well visualized on MRI (Fig. 7). Other complications like mastoid and subperiosteal abscesses, temporal lobe abscesses, and sinus thrombophlebitis are rare and are better evaluated with MRI or alternatively contrast-enhanced MDCT [53].

## Differential diagnosis

**Fig. 8:**
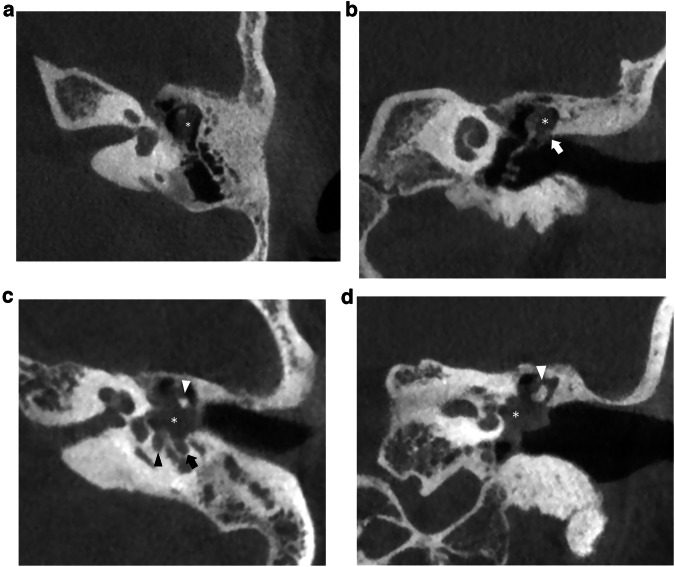
Axial (**a**) and coronal (**b**) CBCT images of a typical pars flaccida cholesteatoma in the left temporal bone extending in Prussak’s space with medial displacement and erosion of the ossicular chain (asterisk in **a**, **b**). Notice the erosion of the scutum (white arrow in **b**) and the presence of a tympanostomy tube. Axial (**c**) and coronal (**d**) CT of a typical pars tensa cholesteatoma (asterisk in **c**, **d**), originating in the lower and posterior part of the middle ear, extending in the tympanic sinus (black arrowhead) and facial recess (black arrow). There is lateral displacement of the ossicular chain and erosion of the incudostapedial joint (white arrowheads in **c**, **d**) which should be visible on this coronal slice. Both these lesions and CC are often undiscernible on MRI

**Fig. 9:**
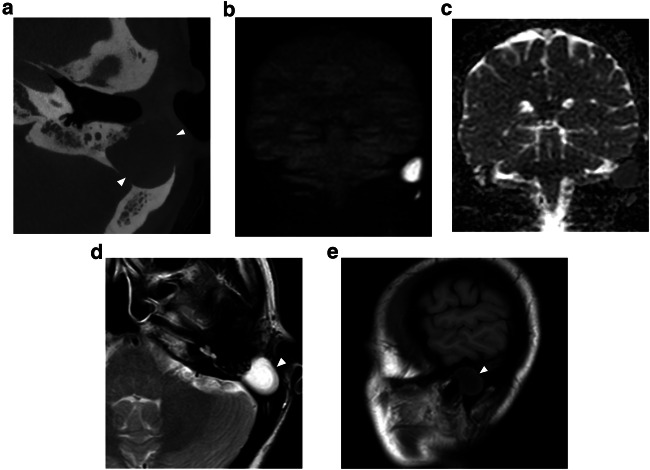
Thirty-eight-year-old women investigated for conductive hearing loss on the right with an incidentally found CC in the mastoid part of the left temporal bone (arrowheads in **a**, **d**, **e**). Note the convex edges, well-defined bone erosions and lack of inflammatory changes in the middle ear on the axial CBCT image (**a**). The MRI shows a typical high signal on DWI (**b**) and low signal on the ADC map (**c**), high signal intensity on T2-WI (**d**) and low signal intensity on T1-WI (**e**), compatible with a CC

**Fig. 10:**
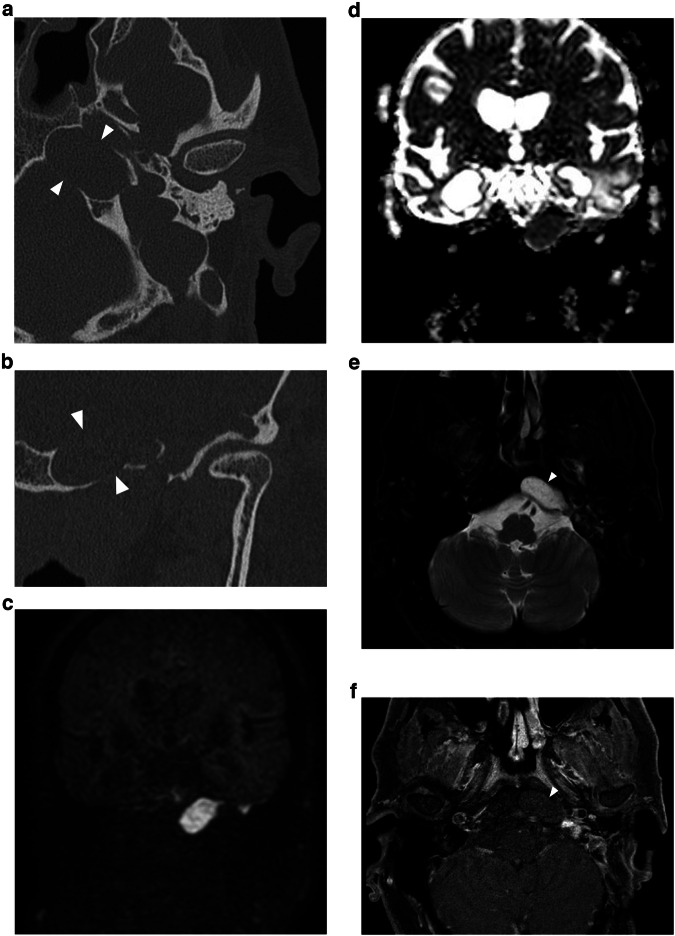
Petrous apex (Class V) CC. **a**, **b** Axial and coronal reformatted CT images of the left temporal bone. (**c**) b1000 DWI, (**d**) ADC map, (**e**) T2-WI with fat suppression and (**f**) T1-WI with fat suppression after intravenous contrast administration of the fossa posterior. There is a well-circumscribed lytic lesion located in the petrous apex (white arrowheads in **a**, **b**, **e**, and **f**). The lesion has typical signal characteristics on MRI with restricted diffusion, excluding inflammatory changes. There is a low signal intensity on T1-WI, excluding a cholesterol granuloma. Case courtesy of Juliano Amy MD, Mass Eye and Ear, Boston, Massachusetts, USA

**Fig. 11:**
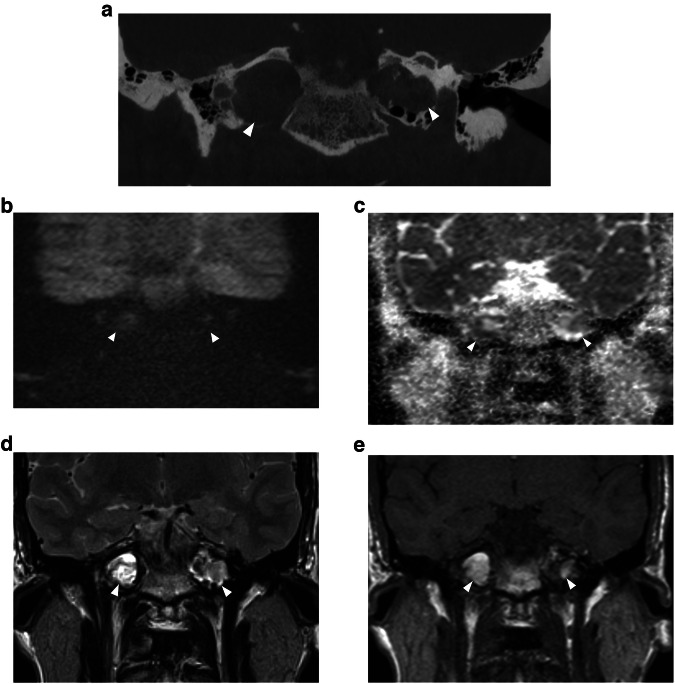
Bilateral cholesterol granuloma of the petrous apex in a 34-year-old man. **a** Coronal reformatted CBCT image showing a sharply delineated lytic lesion in the petrous apex on both sides (white arrowheads). **b** Coronal b1000 DWI, (**c**) ADC map, (**d**) T2-WI and (**e**) T1-WI. The lesions in both the petrous apices (white arrowheads) have a heterogenous signal on DWI and ADC with some signal hyperintensity owing to T2 shine-through. The lesions have an intermediate to high signal intensity on T2-WI. The high signal intensity on T1-WI, however, is almost pathognomonic for a cholesterol granuloma

Differentiation of CMEC from a pars flaccida AC is often easily made. Findings suggesting a pars flaccida AC include a retraction pocket at the pars flaccida of the tympanic membrane with erosion and blunting of the scutum, widening of Prussak’s space, lateral erosion of the incudomalleolar joint, and medial displacement of the ossicular chain (Fig. 8). In larger lesions, however, differentiation can become more difficult. Pars tensa AC on the other hand tend to originate in the lower and posterior half of the tympanic membrane. They often grow posteromedially and superiorly, filling the tympanic sinus and facial recess (Fig. 8). Because these extend medially in the middle ear cavity the differentiation from CC can be more challenging, however, clinical information and visualization of a tympanic perforation and/or retraction pocket often allows to make the correct diagnosis. Other differential diagnoses for CMEC to consider include middle ear adenoma, facial nerve peripheral nerve sheath tumor, tympanic paraganglioma, or an aberrant internal carotid artery [54]. Differentiation is made usually based upon the specific anatomical sites of these lesions, except for middle ear adenoma which is usually diagnosed by excluding all prior entities and its lack of diffusion restriction. Inflammatory changes in the middle ear but also in the mastoid and petrous apex is a common incidental findings but usually do not present a diagnostic challenge. An expansile lesion with convex edges, varying degrees of bone erosions, some preserved cell aeration, and restricted diffusion all favor CC (Fig. 9) [33, 55, 56]. Differential diagnosis of CPBC with other lytic petrous bone lesions is usually straightforward even on CT when considering the location of the lesion and the relatively well-defined erosion. When in doubt MRI can be used to demonstrate restricted diffusion, in keeping with CPBC. Isolated petrous apex cholesteatomas have a specific differential diagnosis including cholesterol granuloma, cephalocele, mucoceles, and some tumoral lesions like chondrosarcoma or plasmacytoma. Cholesterol granuloma is the most common primary petrous apex lesion. On MRI, cholesterol granulomas do not show restricted diffusion. They are typically hyperintense on T2-WI and T1-WI because of related blood breakdown products and proteinaceous contents, making them the only lesions with hyperintense signals on both sequences (Figs. 10, 11) [33, 57].

## Management

### Treatment

Disease eradication and hearing preservation are the primary goals of treatment. Although CC is a benign non-neoplastic process, lesions will eventually spread into different compartments and cause irreversible damage to the surrounding structures of the tympanic cavity and inner ear. As the disease progresses it becomes more difficult to fully eradicate and therefore, timely surgical intervention is mandatory. Preoperative imaging for disease staging is helpful in determining the appropriate approach [34]. The choice between a one-stage procedure and a two-stage procedure with secondary ossicular chain reconstruction depends on disease extension and the surgeon’s confidence in complete disease removal.

Most small CMECs can be removed using a microscope at the meatal opening, a technique called transcanal microscopy. For larger lesions, however, a rigid endoscope inserted into the middle ear offers a significant advantage in capable hands. Endoscopy allows better visualization of the cochleariform process and tensor tympani tendon as well as evaluation of the epitympanic recess and sinus tympani [58]. Disease extension into the mastoid often requires mastoidectomy. The canal wall-down technique was originally developed for full access through the mastoid but it has been replaced by the more patient-friendly canal wall-up surgery wherever possible [59].

CPBC often requires more complicated or combined approaches, especially in cases with disease extension medial to the semicircular canals, petrous apex involvement, or in cases with intracranial complications. Removal of all the temporal bone air cells is often needed to fully expose the lesion. Removal of the semicircular canals or removal of the complete membranous labyrinth can be done to achieve full disease eradication [20, 60]. In such cases, however, hearing and vestibular function are sacrificed. Novel techniques are being developed continuously to preserve vestibular and cochlear functions and improve patient outcomes [61].

When dealing with a CC involving both the middle and inner ear components, it is crucial to ensure the complete removal of both components. Any residual tissue, particularly within the inner ear, can lead to continued growth and recurrence of symptoms.

When the facial nerve function is preoperatively normal, the prognosis is excellent if resection around the nerve is performed gently and under monitoring. In cases where the nerve is more severely damaged patients often present with higher-grade facial nerve palsy and postoperative outcomes are poorer. Facial nerve reanimation can be attempted, often with interposition grafting or hypoglossal-facial nerve anastomosis [60].

### Residual disease and follow-up

**Fig. 12:**
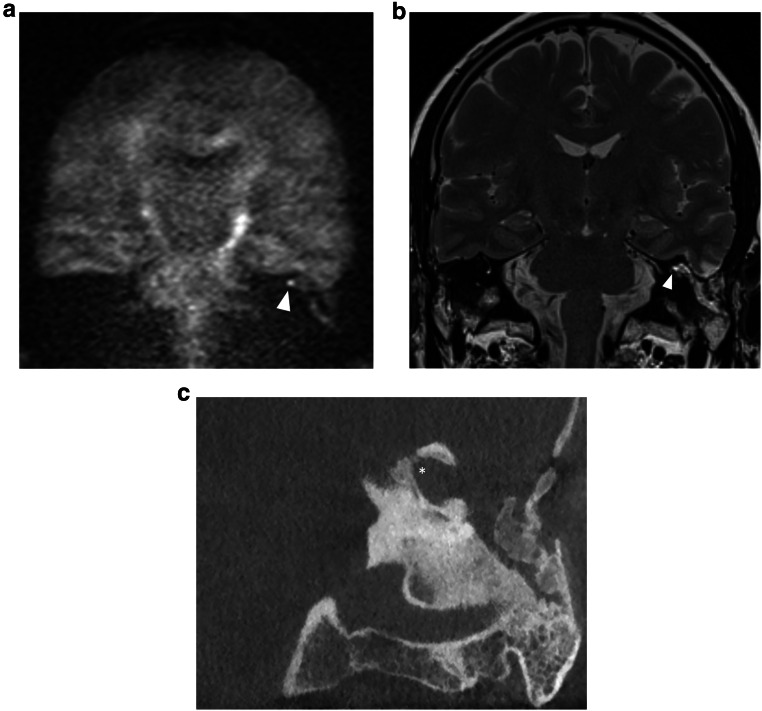
Same case as in Fig. 6, three years after initial diagnosis and surgery. MRI of the posterior fossa shows a focus of restricted diffusion (**a**) with corresponding high signal intensity on the T2-WI (**b**), in keeping with residual CC (arrowheads). **c** CBCT of the temporal bone shows soft tissue filling the mastoid cavity but the residual disease is not discernable (asterisk)

The absolute cornerstones of follow-up are otoscopic examination and MRI with non-EP DWI, often nullifying the need for surgical exploration (Fig. 12). There is no established agreement on when follow-up for CC should occur. In AC the mean time for residual or recurrent disease detection is three years after surgery and radiological follow-up has been proposed at one, three and five years [62, 63]. In addition to clinical follow-up, multiple studies have adopted a similar radiological follow-up for CC [17, 59, 64]. CBCT is often not able to discern residual disease from other etiologies of cavity opacification. CBCT, however, may still be useful for follow-up in a well-aerated middle ear as well as for preoperative planning. Rates of residual or recurrent disease are not higher in congenital compared to AC despite often delayed diagnosis. A possible reason might be the usually absent or less severe dysventilation of the middle ear as a substrate for cholesteatoma formation [58]. Potsic stage III and IV, large lesions (≥ 4 mm), and open-type cholesteatoma have the highest risk of residual disease [17, 24, 65]. When evaluating for residual disease, radiologists should pay special attention to the sinus tympani, stapes footplate, epitympanum, and mastoid cavity [66]. Residual or recurrent disease in petrous bone cholesteatoma is reported to be between 5% and 10% for all etiologies combined [20, 22]. It is important to note that the clinical and radiological follow-up should be tailored to each unique case, considering various negative predictors and the surgeon’s preferences.

## Conclusion

In conclusion, CC represents a fascinating but complex entity. Although it remains relatively rare, increased awareness and imaging advances contribute to a growing incidence. Imaging plays a crucial role in the diagnosis, presurgical planning, and follow-up of CCs and this article provides an overview of state-of-the-art imaging, particularly cone beam CT and non-EP DWI. Treatment strategies vary, balancing disease eradication and hearing preservation. Follow-up protocols, individualized for each case, are crucial for managing potential residual disease.

## Abbreviations

AC: Acquired cholesteatoma
ADC: Apparent diffusion coefficient
CBCT: Cone beam computed tomography
CC: Congenital cholesteatoma
CMEC: Congenital middle ear cholesteatoma
CPBC: Congenital petrous bone cholesteatoma
CT: Computed tomography
DWI: Diffusion-weighted imaging
EP: Echo planar
IAC: Internal auditory canal
MRI: Magnetic resonance imaging
MDCT: Multidetector computed tomography
T1-WI: T1-weighted images
T2-WI: T2-weighted images
